# Simultaneous Acute Cholecystitis and Acute Appendicitis Treated by a Single Laparoscopic Operation

**DOI:** 10.1155/2012/575930

**Published:** 2012-07-08

**Authors:** Jonas P. DeMuro

**Affiliations:** ^1^Division of Trauma & Critical Care, Department of Surgery, Winthrop University Hospital, 259 First Street, Mineola, NY 11501, USA; ^2^Department of Surgery, Stony Brook School of Medicine, 101 Nicolls Road, Stony Brook, NY 11794, USA

## Abstract

While acute appendicitis and acute cholecystitis are both common, they are only rarely seen simultaneously. The clinical presentation and hospital course of a 45-year-old female with concurrent acute appendicitis and acute cholecystitis is presented. The laparoscopic approach is ideal for dealing with multiple, simultaenous abdominal pathologies.

## 1. Introduction

Acute appendicitis and acute cholecystitis are among the most common diagnoses that general surgeons operate on. However, they are only rarely described simultaneously.

## 2. Case Presentation

A 45-year-old female presented to the emergency department with a 1-day history of new onset abdominal pain. The abdominal pain was described as severe and sharp. It localized to the epigastric, right upper, and right lower quadrants as well as radiated posteriorly. She had nausea, as well as multiple episodes of nonbilious vomiting. The past medical history was significant for breast cancer, and the past surgical history was significant for a c-section, bilateral mastectomy, and bilateral tubal ligation.

On admission, her temperature was 97.8°F and hemodynamically stable. Her exam was significant for epigastric tenderness, as well as a positive Murphy's sign, and tenderness over McBurney's point. She had no peritoneal signs, and a negative psoas, obturator, and Rovsing's sign. Clinically, her general appearance was not consistent with sepsis or bacteremia. Her white blood cell count was 8 K/*μ*L with a hematocrit of 37.8%. Her admission chemistry was normal, but her hepatic profile revealed an elevated total bilirubin of 1.4 mg/dL, a direct bilirubin of 0.5 mg/dL, and normal transaminases and alkaline phosphatase. The urinalysis revealed 3+ occult blood.

**Figure 1:**
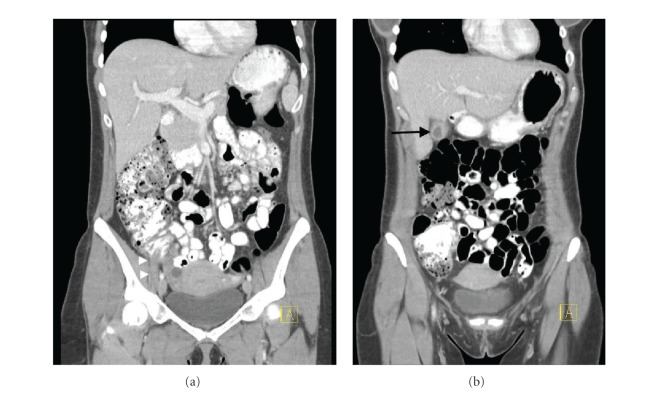
CT abdomen and pelvis, with oral and IV contrast. Note the dilated appendix (white triangles) on the left panel and the inflamed gallbladder (black arrow) on the right panel.

CT abdomen and pelvis with contrast revealed cholelithiasis with the possibility of cholecystitis, as well as a dilated (9 mm) fluid filled appendix suggesting the possibility of early appendicitis (Figure 1). An abdominal sonogram was performed, which confirmed the acute cholecystitis with cholelithiasis with the common bile duct size of 4 mm but did not visualize the appendix.

**Figure 2:**
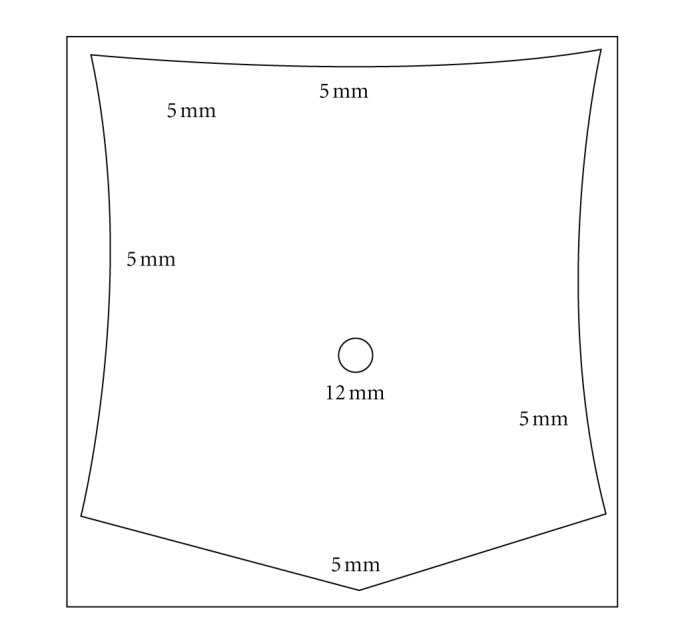
Port placement. The infraumbilical port was placed with the open Hasson technique and all the 5 mm trocars were blunt.

The patient was brought to the operating room where a laparoscopic cholecystectomy and appendectomy was performed. The appendix intraoperatively appeared to be an early appendicitis, nonperforated. The abdomen was accessed via a single 12 mm port and five additional 5 mm ports (Figure 2). The surgery time was 2 hours and 3 minutes. The final pathology revealed both acute and chronic cholecystitis with cholelithiasis, as well as nonperforated acute appendicitis with periappendicitis. The patient was discharged home on postoperative day 2 and made an uneventful and full recovery.

## 3. Discussion

An important principle of medical diagnosis is for an acute onset of symptoms, the constellation of data should fit into a single best diagnosis. However, in this patient, with her abdominal pain prominent in several areas without diffuse peritonitis, clinical suspicion was raised early of the possibility of more than one pathological process. The laparoscopic approach was ideal for this patient as it allowed access to both the appendix and the gallbladder, avoiding multiple open incisions or the morbidity of a full exploratory laparotomy.

Simultaneous appendicitis and cholecystitis in a single patient has only been rarely reported previously. It has been described with an acalculous cholecystitis and appendicitis, [1] as well as during pregnancy [2]. On literature review, only a total of three cases of simultaneous acute appendicitis and acute cholecystitis could be found in the English literature [3]. In the case presented, the occurrence of the two simultaneous pathologies was coincidental, and they were unrelated to each other.

While the overwhelming majority of patients with abdominal pain have a single diagnosis, clinicians need to be aware that multiple diagnoses can rarely coexist. In such cases, a laparoscopic approach can be an ideal approach, allowing surgical access to the entire abdomen.
